# Artemether resistance *in vitro* is linked to mutations in PfATP6 that also interact with mutations in PfMDR1 in travellers returning with *Plasmodium falciparum* infections

**DOI:** 10.1186/1475-2875-11-131

**Published:** 2012-04-27

**Authors:** Dylan R Pillai, Rachel Lau, Krishna Khairnar, Rosalba Lepore, Allegra Via, Henry M Staines, Sanjeev Krishna

**Affiliations:** 1 Department of Pathology and Laboratory Medicine, Medicine, and Microbiology & Infectious Diseases, University of Calgary, Alberta, Canada; 2 Public Health Laboratory, Toronto, Ontario, Canada; 3 Department of Physics, Sapienza University of Rome, Rome, Italy; 4 Centre for Infection and Immunity, Division of Clinical Sciences, St. George’s, University of London, London, SW17 0RE, UK

**Keywords:** Artemisinin resistance, *pfmdr1*, *pfatp6*, Gene copy number, Malaria, Travellers, *Plasmodium*

## Abstract

**Background:**

Monitoring resistance phenotypes for *Plasmodium falciparum*, using *in vitro* growth assays, and relating findings to parasite genotype has proved particularly challenging for the study of resistance to artemisinins.

**Methods:**

*Plasmodium falciparum* isolates cultured from 28 returning travellers diagnosed with malaria were assessed for sensitivity to artemisinin, artemether, dihydroartemisinin and artesunate and findings related to mutations in *pfatp6* and *pfmdr1*.

**Results:**

Resistance to artemether *in vitro* was significantly associated with a *pfatp6* haplotype encoding two amino acid substitutions (*pfatp6* A623E and S769N; (mean IC_50_ (95% CI) values of 8.2 (5.7 – 10.7) for A623/S769 *versus* 623E/769 N 13.5 (9.8 – 17.3) nM with a mean increase of 65%; p = 0.012). Increased copy number of *pfmdr1* was not itself associated with increased IC_50_ values for artemether, but when interactions between the *pfatp6* haplotype and increased copy number of *pfmdr1* were examined together, a highly significant association was noted with IC_50_ values for artemether (mean IC_50_ (95% CI) values of 8.7 (5.9 – 11.6) *versus* 16.3 (10.7 – 21.8) nM with a mean increase of 87%; p = 0.0068). Previously described SNPs in *pfmdr1* are also associated with differences in sensitivity to some artemisinins.

**Conclusions:**

These findings were further explored in molecular modelling experiments that suggest mutations in *pfatp6* are unlikely to affect differential binding of artemisinins at their proposed site, whereas there may be differences in such binding associated with mutations in *pfmdr1*. Implications for a hypothesis that artemisinin resistance may be exacerbated by interactions between PfATP6 and PfMDR1 and for epidemiological studies to monitor emerging resistance are discussed.

## Background

Identifying associations between genotypes of *Plasmodium falciparum* and artemisinin resistance, however it may be defined, is of paramount importance. Attempts to demonstrate clear-cut associations between genotype and phenotype are challenged by variable definitions of clinical treatment failure [1], dormancy in immature parasites that do not show increased *in vitro* resistance to artemisinins but recrudesce after exposure to high drug concentrations [2-4], and the absence of interpretive breakpoints that demarcate susceptibility and resistance to artemisinins.

Several years ago, PfATP6 was hypothesized to be, a SERCA-type calcium pump of the parasite that was a potential target for artemisinins [5]. One implication of this hypothesis was that mutations in PfATP6 might influence susceptibility to artemisinins [6]. Decreased *in vitro* sensitivity to artemisinins was later associated with mutations in *pfatp6* (particularly coding for an S769N substitution) in some geographically and temporally dispersed observations [7-9]. However, there are reports (reviewed in [10]) that do not associate other SNPs in *pfatp6* with decreased susceptibility to artemisinins, perhaps because the detection of these associations may be confounded by the intrinsic polymorphic tendency of this sequence [8,11,12] or lack of detailed phenotypic characterizations. Alternative mechanisms of action and resistance have also been examined for artemisinins (reviewed in [10,13,14]), with these hypotheses having different implications for monitoring of resistance and new drug development programmes. Consequently, any evidence obtained from patient isolates that can clarify relationships between parasite genotypes and artemisinin sensitivity will be useful, particularly as *in vitro* models may not be able to replicate phenotypes of resistance to artemisinins observed in clinical isolates.

Increased copy number for *pfmdr1* is associated in many geographic areas with elevations in IC_50_ values to arylaminoalcohols (mefloquine and lumefantrine) and artemisinins. These observations were first made in laboratory models of drug resistance and confirmed in isolates from patients in Southeast Asia [15-18]. Increased *pfmdr1* copy number is established as a clinically relevant determinant of treatment failure with mefloquine even when given with artesunate [17,19]. Laboratory *in vitro* and *in vivo* models have confirmed the causal link between *pfmdr1* copy number and multidrug resistance [20,21]. Amino acid substitutions in *pfmdr1* may also modulate drug susceptibility in clinical isolates [22,23].

Mechanisms of artemisinin resistance were studied in travellers returning to Southern Ontario with a presumptive diagnosis of *P. falciparum* infection who had blood specimens submitted to the Toronto Public Health Laboratory for confirmation of diagnosis. As part of a study to validate *in vitro* susceptibility testing for *P. falciparum*, parasites were introduced into cultures and their drug sensitivity profiles assessed. An early case report from these efforts suggested that some parasites manifest *in vitro* resistance to the artemisinin class of anti-malarials [24], and that this phenotype could be linked to polymorphisms in *pfatp6*, and *pfmdr1* copy number. This report expands those preliminary findings and provides results from approximately three times the number of isolates originally examined. Here, the hypothesis that particular mutations in *pfatp6* are associated with elevations in IC_50_ values to some artemisinins is tested, and the potential contribution of mutations in *pfmdr1* to this resistance phenotype is examined.

## Methods

### Parasites

Blood specimens were obtained from patients for routine diagnostic purposes and placed into culture for *in vitro* drug susceptibility assays or directly tested using an *ex vivo* assay. Results for some of these parasites (n = 10) have been previously reported [24]. This study was approved by the Ethics Committee of Mount Sinai Hospital (MSH REB #07-0337-E) in Toronto, Ontario.

### Drug susceptibility assays

*Ex vivo* drug susceptibility testing was conducted in triplicate using fresh isolates from blood of patients with microscopy confirmed cases of *P. falciparum*[25]. Isolates were diluted with uninfected blood to 0.05% - 0.2% parasitaemia and 1.5% haematocrit. An aliquot of 195 μL of sample in RMPI 1640 and 0.5% Albumax I (Invitrogen) was loaded into a 96-well plate and 5 μL of drug was added with a Biomek 3000 (Beckman Coulter) automated liquid handler. Anti-malarial drugs, artemisinin (Sigma-Aldrich 361593), artesunate (Sigma-Aldrich A3731), dihydroartemisinin (DHA; SensaPharm RS-004) and artemether (SensaPharm RS-010) were serially diluted 1 in 5 from a stock solution of 250 μM in the series: 6250, 1250, 250, 50, 10, 2, 0.4, 0.08 and 0.016 nM. Plates were frozen for at least 24 h at −80°C after incubation at 37°C, 5%, CO_2_, 5%, O_2_ for 72 h and then thawed and checked for complete lysis of erythrocytes before analysis by HRPII ELISA assay [25]. All plates contained a reference parasite strain for quality control purposes.

*In vitro* drug susceptibility tests were performed in triplicate using cultured *P. falciparum* isolates. Isolates were synchronized to achieve over 70% immature (ring) stages and were diluted with uninfected erythrocytes to 1–1.2% parasitaemia and 0.5% haematocrit. Erythrocytes and drugs were added and incubated as above for 72 h. Erythrocytes were stained with SybrGreen I and analysed by Beckman Coulter FC500 Flow Cytometer.

### Sequence analysis for *pfatp6* and *pfmdr1* and copy number assay

Pyrosequencing, using methodology described previously [26], was used to detect a subset of known SNPs in genes associated with resistance to anti-malarial drugs in *P. falciparum*; such as mutations in codon 623 and 769 of the *P. falciparum* calcium-transporting ATPase gene (*pfatp6,*Genbank ID EF564342.1) previously associated with resistance to artemisinins [7,8]; mutations in codons 86, 184, 1034, 1042, and 1246 of the *P. falciparum* multi-drug resistance protein 1 gene (*pfmdr1*) associated with resistance to multiple anti-malarial drugs [27]. Briefly, biotinylated PCR products, produced using a PyroMark PCR Kit (Qiagen), were purified and captured on streptavidin sepharose beads (GE Healthcare) through a vacuum preparation protocol. They were then resuspended in sequencing primer solution, denatured in a heat block and annealed to the sequencing primer of interest. Forward, reverse and sequencing primers are shown in Additional file 1. Sequencing assays were performed in a PyroMark Q24 (Qiagen), according to the manufacturer’s protocol, and analyzed using the supplied Q24 software. Also *pfmdr1* copy number was determined in combination with the SNPs to study the association between the increase in *pfmdr1* gene copy number and drug resistance [17].

### Statistical analyses

Data were summarized in Excel and analysed in GraphPad (Version 5.04). For the primary multiple comparisons (two-tailed, unpaired Student’s *t*-tests) involving *pfatp6* (A623E/S769N) and *pfmdr1* copy number the statistical significance level was set to *p* < 0.025. Other comparisons are exploratory and presented without correction for multiple comparisons but with data made available (Additional file 2). The assumption of normality was tested with Kolmogorov-Smirnov test. Pearson’s product moment coefficient was used to calculate correlations. To assess if presenting parasitaemia may have influenced results after normalizing in culture, values <0.1% were set to 0.1% for statistical purposes. IC_50_ values were calculated by HN-nonlin v1.1 software [28].

### Modelling and docking studies

The sequence of PfATP6 (UniProt Accession Number: Q5R2K6) was obtained from the UniProt database ([29], accessed 2 March, 2011) and is 1228 amino acids in length. It was modelled, as previously reported in detail [30], and the model deposited in the Protein Model Data Base. It can be retrieved using the following IDs: PM0077298 (PfATP6 in state E1) and PM0077299 (PfATP6 in state E2). Using this model, the effects of the two SNPs that have been studied in this report (A623E and S769N) were examined. The molecular structures of artemether, artemisinin and DHA were retrieved from the Cambridge Structural Database, and docked into PfATP6, as previously described for artemether. Initially, ‘blind docking’ experiments were implemented and these were followed by site-specific docking steps, using AutoDock4.2.

The sequence of PfMDR1 (UniProt Accession Number: P13568) detected PDB:3G5U (chain A) as the best template (sequence identity: 31%, E-value < 10^-10^). 3G5U is the X-ray structure of a *Mus musculus* P-glycoprotein [31], an MDR transporter belonging to the ATP-binding cassette superfamily. ‘Inserts’ that could not be modelled were removed manually. Models were compared with a published model for PfMDR1 [32]. The same computational techniques used for modelling and docking PfATP6 were used to examine PfMDR1.

## Results

Twenty-eight parasites were cultured from travellers returning with clinical symptoms of malaria between April 2008 and January 2011. Of these 28 patients, only three had taken any form of prophylaxis. Presenting parasitaemia from these patients ranged from <0.1% - 28%, with most (25/28) infections acquired in sub-Saharan Africa. All patient information, demographic details, symptoms, parasitaemia, results of individual drug sensitivity assays and mutations determined for each isolate are presented in Additional file 2. Results for IC_50_ values are normally distributed for all artemisinins (p > 0.1). There was no effect on IC_50_ values of presenting parasitaemia or assay method (*in vitro versusex vivo*; p > 0.05 for each artemisinin tested).

### Mutational analysis

Several mutations in *pfatp6* and *pfmdr1* were identified (see Methods). Of the eight mutational types, three *pfmdr1* mutations (S1034C, N1042D and D1246Y) were not common enough (≤2 in the dataset) to allow analysis and are not discussed further. There were 11 (39%) parasites with a *pfatp6* A623E/S769N haplotype identified from eight African countries. Of these isolates, all also contained *pfmdr1* Y184F. Two parasites only carried Y184F and no other mutations in *pfatp6* or elsewhere in *pfmdr1*. Furthermore, 14 (50%) parasites contained *pfmdr1* N86Y and 9 (32%) parasites had an increased copy number for *pfmdr1* that rounded to a value of 2 (as described previously [17]). There was no higher order duplication event (*pfmdr1* copy number ≥ 2.5) detected.

### Sensitivity comparisons between artemisinins

For each drug the mean (95% CI) IC_50_ values were 12.3 (10.1 – 14.5), 5.39 (4.34 – 6.44), 3.62 (2.96 – 4.27) and 10.3 (8.09 – 12.5) nM for artemisinin, artesunate, DHA and artemether, respectively. Results for IC_50_ values for different artemisinins were examined for cross-correlations and are summarized in Table 1. Values were moderately or strongly positively correlated for most comparisons.

**Table 1:** **Pearson’s correlation coefficient (*r)*values (95% CI) for IC**_**50**_**comparisons of artemisinins**

**Drug**	**Artesunate**	**DHA**	**Artemether**
**Artemisinin**	0.47 (0.11 – 0.72) *p* = 0.012	0.42 (0.05 – 0.68) *p* = 0.028	0.65 (0.37 – 0.82) *p* = 0.0002
**Artesunate**		0.66 (0.39 – 0.83) *p* = 0.0001	0.60 (0.29 – 0.79) *p* = 0.0008
**DHA**			0.33 (−0.05 – 0.63) *p* = 0.084

### Genotypes and artemisinin sensitivity

**Figure 1:**
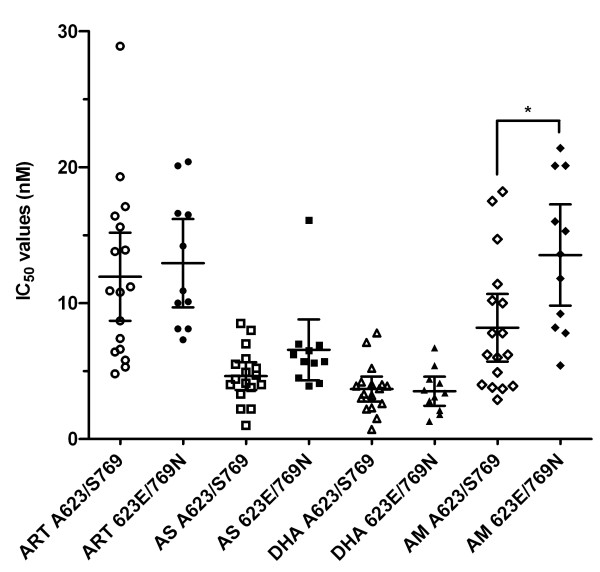
**Association of the *pfatp6 *haplotype (A623E/S769N) and IC**_**50**_**values for artemisinin (ART; circles), artesunate (AS; squares), DHA (triangles) and artemether (AM; diamonds).** Mean individual IC_50_ values are shown for *pfatp6* A623/S769 (open symbols) and *pfatp6* 623E/769 N (closed symbols) containing parasite isolates. The horizontal lines illustrate the mean IC_50_ values for each group. *, *p* = 0.012.

Parasite IC_50_ values for artemisinins were compared between isolates carrying particular mutations (either *pfatp6* A623E/S769N, *pfmdr1* N86Y, Y184F or with increased copy number) and those without. Figure 1 displays the relationship between sensitivity to different artemisinins and *pfatp6* haplotype. Resistance to artemether is significantly increased with mutations in *pfatp6* (mean IC_50_ (95% CI) values of 8.2 (5.7 – 10.7) *versus* 13.5 (9.8 – 17.3) nM with a mean increase of 65%; p = 0.012). Additional file 3 provides 95% CI values for significant differences between means of IC_50_ values of different parasite genotypes.

For parasites with *pfmdr1* Y184F mutations that were present in all A623E/S769N mutants and in 2 additional parasites, differences in artemether sensitivity were attenuated (p = 0.051). However, differences in sensitivity to artesunate became apparent with the inclusion of the two additional parasites with Y184F (mean IC_50_ (95% CI) values of 4.4 (3.4 – 5.4) *versus* 6.5 (4.6 – 8.4) nM with a mean increase of 48%; p = 0.037; see Additional file 3 and Additional file 4A).

Increased *pfmdr1* copy number was not associated with changes in sensitivities to artemisinins (see Additional file 4B). The *pfmdr1* N86Y mutation was associated with increased sensitivity to artemisinin (mean IC_50_ (95% CI) values of 14.7 (10.8 – 18.5) *versus* 10.0 (8.0 – 12.0) nM with a mean decrease of 32%; p = 0.029; see Additional file 2) and DHA (mean IC_50_ (95% CI) values of 4.3 (3.3 – 5.4) *versus* 2.9 (2.1 – 3.7) nM with a mean decrease of 33%; p = 0.020; see Additional file 3 and Additional file 4C).

**Figure 2:**
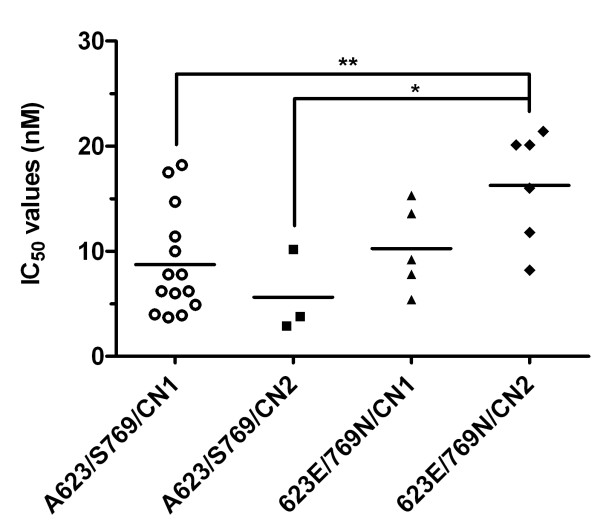
**Association of elevated *pfmdr1 *copy number coupled with the presence or absence of the *pfatp6 *haplotype (A623E/S769N) and IC**_**50**_**values for artemether.** Mean individual IC_50_ values are shown for non-mutant (*pfatp6* A623/S769 and a *pfmdr1* copy number of 1 (CN1), open circles) and mutant parasite isolates containing either *pfatp6* 623E/769 N and a *pfmdr1* copy number of 1 (closed squares), *pfatp6* A623/S769 and a *pfmdr1* copy number of 2 (CN2, closed triangles) or *pfatp6* 623E/769 N and a *pfmdr1* copy number of 2 (closed diamonds). The horizontal lines illustrate the mean IC_50_ values for each group, with 95% CI values of 5.9-11.6, -4.3-15.5, 5.2-15.4 and 10.7 to 21.8 for A623/S769/CN1, A623/S769/CN2, 623E/769 N/CN1 and 623E/769 N/CN2, respectively. **, *p* = 0.0068; *, *p* = 0.02.

The IC_50_ values of artemisinins for parasites carrying pairs of mutations were compared with results from parasites without those mutations when numbers permitted (Figure 2 and Additional file 5). When interactions between the *pfatp6* haplotype and increased copy number of *pfmdr1* were examined together, a highly significant association was noted with IC_50_ values for artemether (mean IC_50_ (95% CI) values of 8.7 (5.9 – 11.6) *versus* 16.3 (10.7 – 21.8) nM with a mean increase of 87%; p = 0.0068; see Additional file 3 and Additional file 5A). A similar significant finding was observed for artesunate (mean IC_50_ (95% CI) values of 5.1 (4.0 – 6.1) *versus* 8.1 (3.9 – 12.2) with an increase of 59%; p = 0.03; see Additional file 3 and Additional file 5A).

*Pfmdr1* N86Y mutations were associated with significantly increased sensitivity to DHA when analysed in isolation of either *pfatp6* A623E/S769N (mean IC_50_ (95% CI) values of 4.5 (3.3 – 5.7) *versus* 2.5 (1.4 – 3.7) nM with a decrease of 44%; p = 0.023; see Additional file 3 and Additional file 5B) or increased *pfmdr1* copy number (mean IC_50_ (95% CI) values of 4.5 (3.3 – 5.7) *versus* 2.6 (1.8 – 3.4) nM with a decrease of 42%; p = 0.0087; see Additional file 3 and Additional file 5C).

### Structural analysis of PfATP6 and PfMDR1

**Figure 3:**
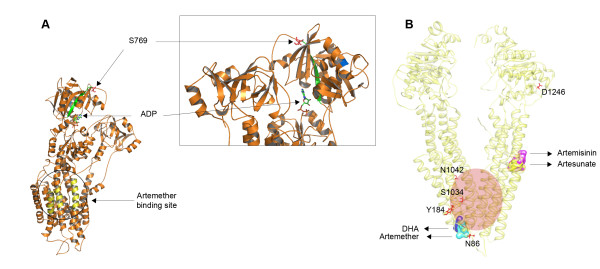
**3D models of (A) PfATP6 and (B) PfMDR1.** (**A**) S769 is highlighted in red. The first (K568) and last (C677) residues flanking the disordered region (which hosts A623) are reported in blue. Notice that the inset presents a zoom of the N domain rotated by 180° (in order to show the position of the disordered insertion, which is not visible in the model on the left). The predicted artemether binding region is in yellow. The beta-strand connecting the ATP-binding site to S769 is in green. (**B**) Blind docking poses for artemisinin, artesunate, DHA, and artemether are presented as filled spheres. SNPs are highlighted in red sticks. The known drug-binding site is shaded in red.

Positions 769 and 623 in PfATP6 occur in the N domain, which is a cytoplasmic putative nucleotide binding domain. In particular, position 623 falls in a long disordered insert, which is unique in PfATP6. These mutations are displayed in Figure 3A (also see Additional files 6 and Additional file 7 for alignments and evolutionary conservation) in the model for PfATP6 and are distant from the predicted artemether binding region [30]. This suggests that any effects of mutation at these positions on the interaction with the drug may be mediated through allosteric mechanisms, which are as yet poorly understood for PfATP6. Comparison between the binding modes of different artemisinin derivatives into the three-dimensional structure of the native PfATP6 suggests that artemether, artemisinin and DHA all interact in a similar way with the proposed binding site.

The possibility that PfMDR1 modulates sensitivities to artemisinins also led to modelling of this sequence and experiments attempting docking with artemisinins. Figure 3B displays a three-dimensional model for PfMDR1 with the known drug-binding site [31] highlighted in light red. Visual inspection of the PfMDR1 model suggested conclusions similar to those of Ferreira *et al*[32]: namely positions 1034 and 1042 are located in the vicinity of the drug-binding site and S1034C and N1042D could in principle have a direct role in the drug binding affinity, whereas SNPs in positions 86 and 184 are more likely to alter drug sensitivity phenotypes by affecting the kinetics of the protein and do not seem to have a direct role in the interaction with the drugs.

Docking experiments on the entire protein (“blind docking”) showed that the drug-binding cavity is preferentially selected by none of the artemisinins (artemisinin, artemether, DHA, artesunate). “Forced docking” of the drugs into the protein region surrounding the drug-binding site (in the red box in Figure 3b) showed that all molecules are in principle able to establish weak interactions with the residues in the binding cavity but no “strong” solutions were found for the native PfMDR1 structure. Docking artemisinin, DHA, and artemether into the mouse MDR1 (MmMDR1) crystal structure (in a ‘blind’ fashion) also found that, in this case, all molecules displayed a clear preference (90/100 docking solutions) for the drug-binding cavity. This observation, together with the finding that wild-type PfMDR1 does not tend to bind artemisinins in such a cavity, suggests that mutations may confer to PfMDR1 the capability of transporting artemisinins either by increasing the drug-binding site affinity to these drugs or by inducing a pleiotropic rearrangement of the helices involved either in the drug access to the cavity or in the transport itself. These mutations may have differential effects on different artemisinin derivatives.

## Conclusions

This report confirms that increased IC_50_ values to artemether are linked to the *pfatp6* A623E/S769N haplotype. There are several reasons why this association may be of interest to those studying mechanisms of drug action and resistance in malaria. Parasites with this *pfatp6* haplotype have originated from dispersed countries in sub-Saharan Africa (Additional file 2). Most have presented without obvious drug selection pressure applied by the traveller, so that it is likely these mutations are present at source. Selection for these polymorphisms may therefore be taking place in the countries of origin for these parasites, by the rapid scale up of anti-malarial treatment programmes being implemented in recent years, and now at about 300 million doses of artemisinin combination therapies disbursed in a year [33,34].

These findings have implications for epidemiological studies monitoring drug resistance to artemisinins. In PfATP6, A623E and S769N substitutions have been reported individually as being associated with elevated IC_50_ values to artesunate and artemether respectively [7,35]. Each of these polymorphisms has also been associated with other amino acid substitutions in PfATP6, but not hitherto with each other (reviewed in [10]). Combinations of mutations in PfATP6 may result in more obvious effects on IC_50_ values, with the haplotype observed in these studies emerging as being an important one. Despite the highly polymorphic nature of PfATP6 [8,12], the independent linkage between artemether and mutations described here confirms previous observations from French Guiana that these mutations are worth monitoring in future epidemiological studies of artemisinin resistance [7]. Other reported mutations may also prove epidemiologically useful once the intrinsic variability of PfATP6 sequence can be distinguished from those that have relevance to artemisinin sensitivity, as discussed in detail elsewhere [12].

The magnitude of elevation in IC_50_ values for artemether in PfATP6 mutant-bearing parasites is approximately two-fold (Figure 1 and Results). This difference is larger than between parasites carrying the L263E mutation in PfATP6 when compared with control parasites (23%), for artemisinin and DHA [36]. Interestingly, increases in IC_50_ values for L263E mutants did not apply to all the artemisinins examined (for example, artesunate) and this also is the case in this study, where artemether is the derivative predominantly affected by these mutations in PfATP6 (Figures 1 and 2). Mutations in PfATP6 identified in isolates taken from patients (in contrast to L263E) may not fall within protein areas of obvious functional significance for SERCA type activity that have been identified in mutagenesis studies over several decades in many organisms, and by more recent solutions of crystal structures [37-39]. They are located in less highly conserved regions, so this may reflect ignorance of structure-function relationships in polymorphic plasmodial proteins that contain low complexity ‘inserts’, as suggested almost two decades ago [40]. These studies also illustrate some difficulties in developing laboratory models of drug resistance with transgenic parasites because resistance phenotypes may be difficult to identify in some contexts. For example, resistance observed with K76T *pfcrt* for chloroquine, may depend on genetic context [41]. Part of this genetic context includes the *pfmdr1* gene, which itself can modulate *in vitro* sensitivity to a variety of unrelated drug classes (reviewed in [42]).

Duplication in *pfmdr1* has emerged as being perhaps surprisingly common in these returning travellers. Previous reports from African countries have only demonstrated sporadic instances of *pfmdr1* gene duplication. Duplications can arise within the host after drug treatment as well as being selectable *in vitro* ([20,43,44] suggesting that the potential for this genetic event to occur is high. This is borne out by the correspondingly high prevalence of *pfmdr1* duplications in parasites in countries that have used arylaminoalcohols such as mefloquine as part of their anti-malarial treatment regimens (*e.g.*[17]) as well as the ease for selection of increased copy number in African parasites [45].

There may be a strong association between duplications in *pfmdr1* and mutations in *pfatp6* that influence the IC_50_ values for artemether (and artesunate), because even in a relatively small subset of parasites, there is a highly significant link between mutations in both transporter genes and IC_50_ values. This association is not apparent with *pfmdr1* duplications alone in this relatively small dataset, although it has been noted before in larger studies of field isolates in different regions of the world (*e.g.*[17]), and also in laboratory models [21,46-48].

Several studies have reported on mutations in *pfatp6* and *pfmdr1* gene duplications or polymorphisms as part of epidemiological monitoring for drug resistance, including for resistance to artemisinins. Interestingly, some studies may only be able to provide useful information when parasites are cultured for the short term *ex vivo* because of mixed populations of parasites with and without mutations in *pfatp6* and associated fitness costs of mutations. These can result in disappearance of *pfatp6* mutations after adaptation to continuous culture, as carefully documented previously [9]. Artemisinin or artesunate IC_50_ values are almost double in field isolates from Southeast Asia with gene duplications, whereas in these studies there is no significant change in IC_50_ values to DHA [17,49,50]. Mutations in the 3’ sequence of *pfmdr1* (with D1246Y being a good example) increase sensitivity to artemisinin [51-53], whereas they may affect artesunate in the opposite way, by decreasing sensitivity in field isolates [17]. One of the strongest associations is between the N86Y substitution and increased sensitivity to artemisinin and DHA in this analysis. This finding is consistent with previous reports that this mutation in *pfmdr1* may modulate the effects of artemisinins.

PfMDR1 is localized to the food vacuole of parasites, and studies after heterologous expression suggest that SNPs alter substrate specificity for aminoquinolines and arylaminoalcohols [54] with the suggestion that drugs are removed from the cytoplasm into the food vacuole (reviewed in [55]). Mutations in PfMDR1 may alter IC_50_ values to artemisinins by modulating their removal (to the food vacuole) away from their proposed target (PfATP6 localised in the ER). A simple hypothesis that highlights the importance of interactions between PfATP6 and PfMDR1 in modulating artemisinin sensitivity is that resistant parasites carrying mutations in PfATP6 can become sensitive if SNPs in PfMDR1 decrease delivery of artemisinins to the food vacuole. This suggestion is consistent with results from modelling suggesting that mutations in PfATP6 are unlikely to differentially affect interactions with different artemisinins and PfATP6, whereas PfMDR1 mutations may have variable effects on transport of artemisinins.

These insights into the biology of drug resistance mechanisms have developed from an analysis that combines results from field isolates with contributions from molecular studies including modelling. Studies of returning travellers as ‘sentinels’ for drug resistance may be particularly useful, as previously *pfmdr1* gene duplication in Africa was first recorded in a returning traveller [56]. For epidemiological purposes, monitoring for mutations in both transporter genes (*pfatp6* and *pfmdr1*) should be carried out and related to *in vitro* sensitivity profiles of the clinically relevant artemisinin derivatives.

## Competing interests

The authors declare that they have no competing interests.

## Authors’ contributions

DRP, RL and KK derived the dataset. AV and RL undertook the modelling studies. HMS undertook statistical analyses and helped daft the text. SK conceived the study and drafted the text. All authors read and approved the final manuscript.

## Supplementary Material

Additional file 1 Pyrosequencing primers Click here for file

Additional file 2 Patient information, demographic details, symptoms, parasitemia, results of individual drug sensitivity assays and mutations determined for 28 P. falciparum isolates collected from returning travellers. Excel file Click here for file

Additional file 3 Mean differences and 95% CI of mean differences between comparisons of IC50 values that are significantly associated with particular parasite genotypes (see Results and Additional files 4 and 5) Click here for file

Additional file 4 Association of pfmdr1 haplotypes, Y184F (A), N86Y (B) and copy number (CN1/2; C) and IC50 values for artemisinin (ART; circles), artesunate (AS; squares), DHA (triangles) and artemether (AM; diamonds). Mean individual IC50 values are shown for non-mutant (open symbols) and mutant (closed symbols) containing parasite isolates. The horizontal lines illustrate the mean IC50 values for each group. *, *p* < 0.05 Click here for file

Additional file 5 Association of (A) pfatp6 haplotype at 623/769 versus pfmdr1 copy number (CN), (B) pfatp6 haplotype at 623/769 versus pfmdr1haplotype at 86 and (C) pfatp6 haplotype at 623/769 versus pfmdr1 haplotype at 86 and IC50 values for artemether (ART), artesunate (AS), DHA and artemether (AM). Mean individual IC50 values are shown for non-mutant (open circles) and mutant parasite isolates containing either single mutation (closed squares and triangles) or both (closed diamonds). The horizontal lines illustrate the mean IC50 values for each group. **, *p* < 0.01; *, *p* < 0.05 Click here for file

Additional file 6 Sequence alignments of PfATP6 with the human, rabbit, and schistosomal homologues. The two positions at which SNPs were observed (623 and 769) are highlighted with red-filled circles and the artemether binding regions are in grey boxes Click here for file

Additional file 7 Multiple sequence alignments of the ATP N-domain of PfATP6 with its homologues.The top part of the alignment presents the conservation level along the PfATP6 sequence observed in PfATP6 variants. The bottom part of the alignment presents the conservation level along the PfATP6 sequence observed with its homologues. The higher the column, the more conserved a residue is in the corresponding position. Columns are coloured according to the residue’s chemical properties: cysteines in yellow, aliphatic hydrophobics (V, L, I, M) in green, aromatic amino acids (Y, F, W) in dark green, small amino acids (G, A, S, T) in light grey, negatively charged (D, E) in blue, polar (N, Q) in magenta, histidines in orange, positively charged (K, L) in red, prolines in grey. The black bar in the middle shows the column score between the query sequence and the homologous amino acid distributions with |, +, ., -, = indicating scores from 'very good' to 'very bad'. The ATP binding residue positions are indicated with red arrows and positions 623 and 769 with black arrows. The insertions unique to P. falciparum can be easily recognized where the alignment lacks the bottom part and the black bar (the homologue information Click here for file
