# Supplementary material for: Artemether resistance in vitro is linked to mutations in PfATP6 that also interact with mutations in PfMDR1 in travellers returning with Plasmodium falciparum infections
Source: Malar J. 2012 Apr 27;11:131. doi: 10.1186/1475-2875-11-131 (PMC3422158; doi:10.1186/1475-2875-11-131)
Supplement: Additional file 1 — Pyrosequencing primers [file 1475-2875-11-131-S1.doc]

**Additional file 1: Pyrosequencing primers**

| **SNP** | **Primer sequences (5’ to 3’)^a^** | | |
| --- | --- | --- | --- |
|  | **Forward** | **Reverse** | **Sequencing** |
| *pfatp6* 623 | **CTCAGGCAACAACAAATGGATATG** | ATTACCCAATTTTGAGTGGAAACA | TTCAAAACTTGTGCCA |
| *pfatp6* 769 | **CAAAATATGGGAAAAAGAGCATTA** | TCTGGCCGTATTAATATTATCACC | ATAAATTAAATCTTGTTCTA |
| *pfmdr1* 86 | CGTTTAAATGTTTACCTGCACAA | **TTGTCCATCTTGATAAAAAACACT** | GTGTAATATTAAAGAACATG |
| *pfmdr1* 184 | AGTTCAGGAATTGGTACGAAATTT | **AACGTGCATTTTTTATTAATGACC** | CCAGTTCCTTTTTAGGTT |
| *pfmdr1* 1034 | GCGGAGTTTTTGCATTTAGTTCA | **TTAAGAAGGATCCAAACCAATAGG** | GCAGCTTTATGGGGAT |
| *pfmdr1* 1042 | GCGGAGTTTTTGCATTTAGTTCA | **TTAAGAAGGATCCAAACCAATAGG** | GCAGCTTTATGGGGAT |
| *pfmdr1* 1246 | **TTTTCAAACCAATCTGGATCTG** | CGTTTAACATCTTCCAATGTTGC | ATTGAAAATAAGTTTCTAAG |

^a^ Biotinylated primers (in bold) were HPLC purified, while others were desalted (IDT, Iowa)
