# Supplementary material for: Artemether resistance in vitro is linked to mutations in PfATP6 that also interact with mutations in PfMDR1 in travellers returning with Plasmodium falciparum infections
Source: Malar J. 2012 Apr 27;11:131. doi: 10.1186/1475-2875-11-131 (PMC3422158; doi:10.1186/1475-2875-11-131)
Supplement: Additional file 3 — Mean differences and 95% CI of mean differences between comparisons of IC50 values that are significantly associated with particular parasite genotypes (see Results and Additional files 4 and 5) [file 1475-2875-11-131-S3.docx]

**Additional file 3 Mean differences and 95% CI of mean differences between comparisons of IC_50_ values that are significantly associated with particular parasite genotypes (see Results and Additional files 4 and 5).**

| **Drug** | **Parasite genotype** | **Mean difference (nM)** | **95% CI of mean difference (nM)** |
| --- | --- | --- | --- |
| Artemether | *pfatp6* A623/S769 vs 623E/769N | -5.3 | -9.4 to -1.3 |
| Artesunate | *pfmdr1* Y184 *vs*. 184F | -2.1 | -4.1 to -0.1 |
| Artemisinin | *pfmdr1* N86 *vs*. 86Y | 4.6 | 0.5 to 8.7 |
| DHA | *pfmdr1* N86 *vs*. 86Y | 1.5 | 0.2 to 2.7 |
| Artesunate | *pfatp6* A623/S769/*pfmdr1* CN1 vs *pfatp6* 623E/769N/*pfmdr1* CN2 | -3.0 | -5.6 to -0.3 |
| Artesunate | *pfatp6* A623/S769/*pfmdr1* CN1 vs *pfatp6* A623/S769/*pfmdr1* CN2 | 2.5 | 0.1 to 5.0 |
| Artesunate | *pfatp6* A623/S769/*pfmdr1* CN2 vs *pfatp6* 623E/769N/*pfmdr1* CN1 | -2.2 | -4.4 to -0.1 |
| Artemether | *pfatp6* A623/S769/*pfmdr1* CN1 vs *pfatp6* 623E/769N/*pfmdr1* CN2 | -7.5 | -12.7 to -2.4 |
| Artemether | *pfatp6* A623/S769/*pfmdr1* CN2 vs *pfatp6* 623E/769N/*pfmdr1* CN2 | -10.6 | -18.9 to -2.3 |
| Artemisinin | *pfatp6* 623E/769N/*pfmdr1* N86 vs *pfatp6* 623E/769N/*pfmdr1* 86Y | 7.6 | 3.3 to 12.0 |
| Artemisinin | *pfatp6* 623E/769N/*pfmdr1* N86 vs *pfatp6* A623/S769/*pfmdr1* 86Y | 7.9 | 2.6 to 13.2 |
| DHA | *pfatp6* A623/S769/*pfmdr1* N86 vs *pfatp6* A623/S769/*pfmdr1* 86Y | 1.9 | 0.3 to 3.6 |
| Artemether | *pfatp6* 623E/769N/*pfmdr1* N86 vs *pfatp6* A623/S769/*pfmdr1* 86Y | 9.8 | 3.5 to 16.0 |
| Artemether | *pfatp6* A623/S769/*pfmdr1* 86Y vs *pfatp6* 623E/769N/*pfmdr1* 86Y | -6.6 | -10.6 to -2.6 |
| DHA | *pfmdr1* CN1/86N vs *pfmdr1* CN1/86Y | 1.9 | 0.6 to 3.3 |

CN, copy number
