# Supplementary material for: Artemether resistance in vitro is linked to mutations in PfATP6 that also interact with mutations in PfMDR1 in travellers returning with Plasmodium falciparum infections
Source: Malar J. 2012 Apr 27;11:131. doi: 10.1186/1475-2875-11-131 (PMC3422158; doi:10.1186/1475-2875-11-131)
Supplement: Additional file 4 — Association of pfmdr1 haplotypes, Y184F (A), N86Y (B) and copy number (CN1/2; C) and IC50 values for artemisinin (ART; circles), artesunate (AS; squares), DHA (triangles) and artemether (AM; diamonds). Mean individual IC50 values are shown for non-mutant (open symbols) and mutant (closed symbols) containing parasite isolates. The horizontal lines illustrate the mean IC50 values for each group. *, p < 0.05 [file 1475-2875-11-131-S4.docx]

**Additional file 3: Association of *pfmdr1* haplotypes, Y184F (A), N86Y (B) and copy number (CN1/2; C) and IC_50_ values for artemisinin (ART; circles), artesunate (AS; squares), DHA (triangles) and artemether (AM; diamonds).** Mean individual IC_50_ values are shown for non-mutant (open symbols) and mutant (closed symbols) containing parasite isolates. The horizontal lines illustrate the mean IC_50_ values for each group. *, p < 0.05.
