# Supplementary material for: Artemether resistance in vitro is linked to mutations in PfATP6 that also interact with mutations in PfMDR1 in travellers returning with Plasmodium falciparum infections
Source: Malar J. 2012 Apr 27;11:131. doi: 10.1186/1475-2875-11-131 (PMC3422158; doi:10.1186/1475-2875-11-131)
Supplement: Additional file 5 — Association of (A) pfatp6 haplotype at 623/769 versus pfmdr1 copy number (CN), (B) pfatp6 haplotype at 623/769 versus pfmdr1haplotype at 86 and (C) pfatp6 haplotype at 623/769 versus pfmdr1 haplotype at 86 and IC50 values for artemether (ART), artesunate (AS), DHA and artemether (AM). Mean individual IC50 values are shown for non-mutant (open circles) and mutant parasite isolates containing either single mutation (closed squares and triangles) or both (closed diamonds). The horizontal lines illustrate the mean IC50 values for each group. **, p < 0.01; *, p < 0.05 [file 1475-2875-11-131-S5.doc]

**Additional file 5: Association of (A) *pfatp6* haplotype at 623/769 *versus* *pfmdr1* copy number (CN), (B) *pfatp6* haplotype at 623/769 *versus* *pfmdr1*haplotype at 86 and (C) *pfatp6* haplotype at 623/769 *versus* *pfmdr1* haplotype at 86 and IC_50_ values for artemether (ART), artesunate (AS), DHA and artemether (AM).** Mean individual IC_50_ values are shown for non-mutant (open circles) and mutant parasite isolates containing either single mutation (closed squares and triangles) or both (closed diamonds). The horizontal lines illustrate the mean IC_50_ values for each group. **, p < 0.01; *, p < 0.05.
