# Supplementary material for: Artemether resistance in vitro is linked to mutations in PfATP6 that also interact with mutations in PfMDR1 in travellers returning with Plasmodium falciparum infections
Source: Malar J. 2012 Apr 27;11:131. doi: 10.1186/1475-2875-11-131 (PMC3422158; doi:10.1186/1475-2875-11-131)

# Additional file 6 Sequence alignments of PfATP6 with the human, rabbit, and schistosomal homologues. The two positions at which SNPs were observed (623 and 769) are highlighted with red-filled circles and the artemether binding regions are in grey boxes.

**Cation_ATP**

**E1-E2_ATPase**

**Hydrolase**

**Cation_ATP**


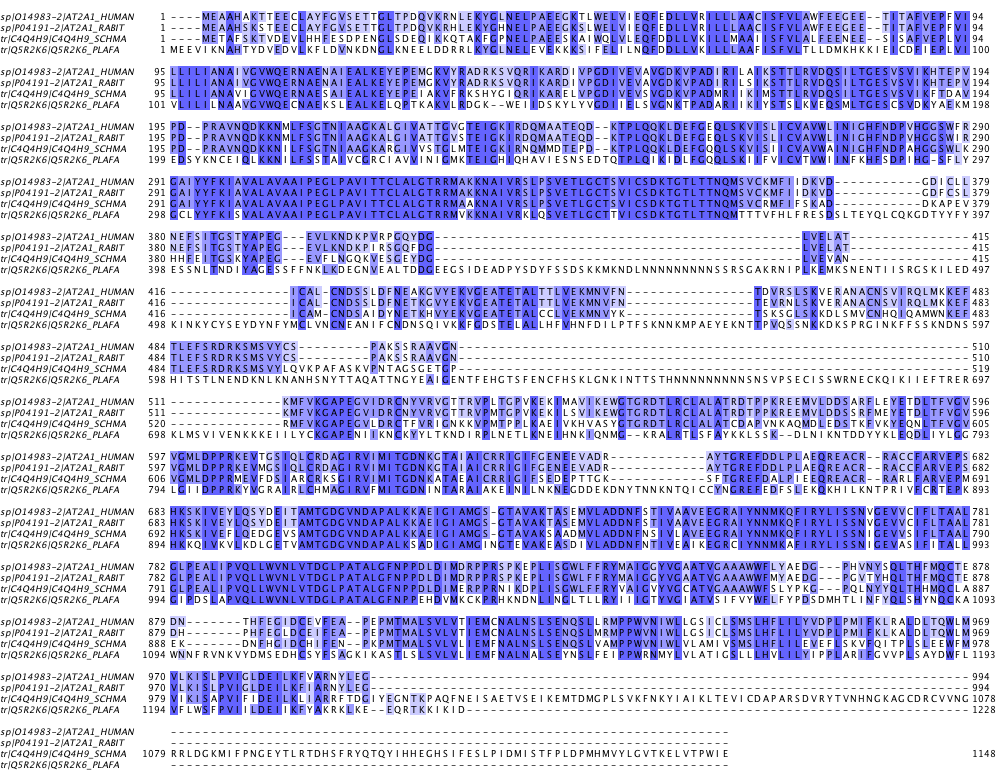

Supplement: Additional file 6 — Sequence alignments of PfATP6 with the human, rabbit, and schistosomal homologues. The two positions at which SNPs were observed (623 and 769) are highlighted with red-filled circles and the artemether binding regions are in grey boxes [file 1475-2875-11-131-S6.doc]
