# Supplementary material for: Artemether resistance in vitro is linked to mutations in PfATP6 that also interact with mutations in PfMDR1 in travellers returning with Plasmodium falciparum infections
Source: Malar J. 2012 Apr 27;11:131. doi: 10.1186/1475-2875-11-131 (PMC3422158; doi:10.1186/1475-2875-11-131)
Supplement: Additional file 7 — Multiple sequence alignments of the ATP N-domain of PfATP6 with its homologues.The top part of the alignment presents the conservation level along the PfATP6 sequence observed in PfATP6 variants. The bottom part of the alignment presents the conservation level along the PfATP6 sequence observed with its homologues. The higher the column, the more conserved a residue is in the corresponding position. Columns are coloured according to the residue’s chemical properties: cysteines in yellow, aliphatic hydrophobics (V, L, I, M) in green, aromatic amino acids (Y, F, W) in dark green, small amino acids (G, A, S, T) in light grey, negatively charged (D, E) in blue, polar (N, Q) in magenta, histidines in orange, positively charged (K, L) in red, prolines in grey. The black bar in the middle shows the column score between the query sequence and the homologous amino acid distributions with |, +, ., -, = indicating scores from 'very good' to 'very bad'. The ATP binding residue positions are indicated with red arrows and positions 623 and 769 with black arrows. The insertions unique to P. falciparum can be easily recognized where the alignment lacks the bottom part and the black bar (the homologue information [file 1475-2875-11-131-S7.doc]

# Additional file 7: Multiple sequence alignments of the ATP N-domain of PfATP6 with its homologues. The top part of the alignment presents the conservation level along the PfATP6 sequence observed in PfATP6 variants. The bottom part of the alignment presents the conservation level along the PfATP6 sequence observed with its homologues. The higher the column, the more conserved a residue is in the corresponding position. Columns are coloured according to the residue’s chemical properties: cysteines in yellow, aliphatic hydrophobics (V, L, I, M) in green, aromatic amino acids (Y, F, W) in dark green, small amino acids (G, A, S, T) in light grey, negatively charged (D, E) in blue, polar (N, Q) in magenta, histidines in orange, positively charged (K, L) in red, prolines in grey. The black bar in the middle shows the column score between the query sequence and the homologous amino acid distributions with |, +, ., -, = indicating scores from 'very good' to 'very bad'. The ATP binding residue positions are indicated with red arrows and positions 623 and 769 with black arrows. The insertions unique to *P. falciparum* can be easily recognized where the alignment lacks the bottom part and the black bar (the homologue information).


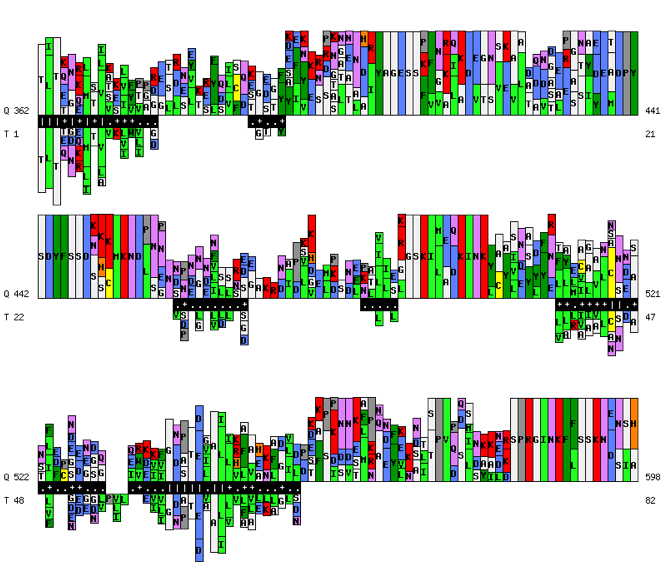


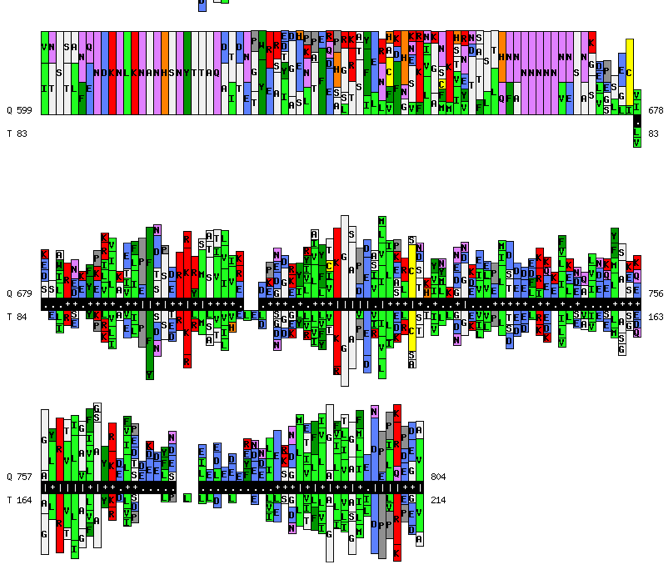


S769

A623
